# Results of Clinical Observation in the Royal Polyclinical Institute of the University of Berlin

**Published:** 1847-07

**Authors:** 


### Art. III.

*Klinische Ergebnisse gesammelt in dem Königlichen Policlinischen Institut der Universität,* von dessen Assistenarzte Dr. Eduard Henoch, und herausgegeben von Dr. M. H. Romberg, &c. &c.—*Berlin,* 1846.

*Results of Clinical Observation in the Royal Polyclinical Institute of the University of Berlin.* Recorded by Dr. E. Henoch, and edited by Dr. Romberg.—*Berlin,* 1846. 8vo. pp. 282.

The results of medical art, as delineated in the present volume, presents points of interest. The name of the editor is a guarantee for the accuracy of the details, and stamps a value upon the clinical and practical information imparted. A field of action, such as the policlinic of a large city like Berlin, must present ample scope for therapeutical experience and experiment, and the practised eye of Dr. Romberg would not fail to view it aright. Further, the preface of the editor contains proofs, that he rightly appreciates the importance of his position as clinical instructor and the value of dispensary practice as a means of instruction.

The cases treated in the dispensary are arranged methodically. First come diseases of the nervous system, according to the neurology adopted by Dr. Romberg, (vide our review of his work in vol. XVII of this Journal): then follow in succession, diseases of the blood, of the alimentary canal, of the sexual and urinary organs, of the thoracic viscera and of the skin.

*Cardiac neuralgia,* or *angina pectoris,* was observed only once; it occurred in a tailor aged 40, and was complicated with disease of the aortal valves: death occurred suddenly. The pain was much relieved by small doses of nitrate of silver.

*Hemicrania* was also successfully treated by the same remedy.

Cases of *chorea* St. Viti were frequent. Nine girls, one boy, and two women, aged 24 and 48 respectively, were treated. The left side only was affected in one case; the right side only in three; and in the eight remaining, both sides. The treatment of the cases, in which intestinal worms were the apparent cause of the disease, consisted in the administration of calomel and jalap: when no causal indication was manifest, the infusion of senna was given first to operate on the bowels, and then chalybeates. The hydrochlorate of iron was found to be the most successful, and was prescribed for children in three-grain doses thrice a day, combined with powdered rhubarb. The arseniate of potass was however found to be the most efficacious remedy: a case of eight years' duration in a girl aged 11 years, and another of 3 years, are reported as recovering under its use. Dr. Romberg began with a dose of two drops of the arsenical solution, gradually increasing to six drops.

*Infantile meningitis* was ushered in by various symptoms. Convulsions, not unlike eclampsia or epilepsy, was one of these. A boy, aged 4 years, had convulsive paroxysms for three days, with intervals of perfect health. On the fourth and fifth days, he did not suffer at all; on the sixth, the convulsions reappeared, accompanied by the usual symptoms of meningitis. A girl, aged 8 years, in perfect health, was attacked with such violent convulsions, that they were pronounced to be epileptic by the practitioner called in; she recovered perfectly, but in twelve hours meningitis set in, and proved fatal in six and thirty hours. In another case, the convulsions preceded the attack of meningitis fourteen days. Dr. Romberg observes, that the condition of the nasal mucous membrane constitutes a good diagnostic sign in these cases. If the secretion be arrested, and the nose be dry, then meningitis is impending. Other signs of meningitis in infants are, the continual attempt to bury the occiput in the pillow, and a frequent change of colour in the cheeks, and especially in one cheek only. Vomiting was often observed, but with these peculiarities, that it occurred when the stomach was empty, without nausea, without straining, and was brought on by moving or shaking the head. The expression of the eyes was noticeable. Children usually follow with their eyes the eyes of their attendants; in meningitis, they cease to do this.

In the majority of cases, the disease was tuberculous meningitis, and was accompanied by deposit of tubercle in other organs, particularly the lungs, bronchial glands, liver, spleen, and mesentery. In the brain, the deposit usually occupied the sulcous folds of the pia mater.

The cases of meningitis infantum were treated after a rigorous antiphlogistic method. Leeches were applied behind the ears, and large doses of calomel given so as to act briskly on the bowels. Cold was applied to the head after the plan recommended by Heim. The child's head is wrapped round the temples with woollen, so that the water may be prevented running over the face, and then cold water is poured upon the head, from the height of one or two feet, for a quarter of an hour. After an interval, the process is repeated.

Several cases of *epilepsy* were treated. Some of these displayed remarkable periodic phenomena. A boy at the age of 9 years, had an attack of epilepsy brought on by fright, the fit recurring every evening at a certain hour. It disappeared without any treatment being adopted. In his twelfth year, he fell on the ice while skating, having a headache and other cerebral symptoms at the time; and immediately he was again subject to periodic epileptic fits, recurring every night at ten or eleven o'clock. In another case, a young woman, the fits recurred every evening about five o'clock. In another case, the paroxysm recurred every month, about the time of the new moon; the whole paroxysm or attack continued twenty-four hours, and was made up of twelve fits, having an interval of coma of two hours' duration between each. In a note Dr. Henoch mentions the case of a young gentleman who had a fit with the greatest regularity every seventh day, from eight to half-past eight o'clock in the morning.

*Paraplegia.* The suppression of the perspiration of the feet is mentioned by Romberg as a cause of this disease. We are inclined to doubt the accuracy of the etiological views given by him, as it seems more probable that the suppression of the perspiration was rather a coincident than an antecedent. A healthy man, aged 46, who had been troubled from his youth with profuse perspiration of the feet, had suffered for two years past from frequent pains in the limbs, which were worse towards night, were evidently of a rheumatic character, and had followed his getting cold and wet in the feet. The perspirations at last were suddenly suppressed, and immediately after the patient experienced a feeling of weakness and loss of sensibility in the lower extremities. At first, these phenomena were confined to the leg, but extended subsequently to the thigh and lower abdomen, and were accompanied by eneuresis and constipation. Totally unable to walk, he was able to move about on crutches, dragging the lower half of his body after him; but when seated, he could move his legs in any direction. Perspiration was limited to the upper half of his body, ending just below the umbilicus. The lower half was dry, and desquamated continually. Sexual power was lost. The most careful exploration of the spinal column was instituted, but nothing could be discovered to account for the paraplegia. Dr. Romberg then conceived, that the suppression of the habitual perspiration from the feet at least indicated the therapeia in the case, and he prescribed the foot-bath, with half an ounce of "aqua regia" (nitro-muriatic acid) thrice a week. In a fortnight, the gait became steadier, the patient was able to stand, the feet hitherto cold and dry, became warm and moist. From gr. $\frac{1}{3}$ to gr. $\frac{1}{2}$ of the spirituous extract of nux vomica was then administered, and in fourteen days the urinary bladder recovered its tone, and sensation returned to the lower extremities. After a few months' perseverance in the treatment, the patient recovered perfectly, except a slight tottering in his gait.

Another case, somewhat similar, was observed in the person of a drunkard. In this, the bladder and rectum were not paralysed, nor was sensation lost, and the patient could move his legs when seated, as in the preceding case. Upon inquiry it was found, that a profuse habitual perspiration from the feet disappeared immediately previously to the accession of the paralysis. Foot-baths, thrice a week, medicated with caustic potass instead of acid, and continued for a month, effected a cure without the use of any other remedies.

*A new form of atrophy of the face.* This case is interesting, as an example of hemiplegic *atrophy* only. A woman, aged 28, had, at the age of 13, an attack of tertian ague. Three years afterwards, she made a journey on foot through a very heavy rain, and very soon after was attacked by what was probably scarlet fever. In particular, the mucous membrane of the pharynx was inflamed, the voice was hoarse, deglutition painful, the uvula enlarged, and an abscess formed in the left tonsil. Externally, the cervical glands were swollen. The general health was shortly re-established, but it was found, that the right side of the face appeared full, plump, and blooming, as it should be in a female of eight and twenty, while the left side was shrivelled and wrinkled like that of an old woman. The median line of the face constituted a sharp and distinct boundary between youth on the one side of the face and age on the other. On the wrinkled side, the hair was scanty, and the forehead less convex; but from the want of muscle and fat, the superciliary arch on that side was more strongly marked than on the right. Further, the eyebrow was nearly all gone, the eyelashes scanty, the caruncula small and pale, the eyelids large and thin, as compared with the right. The left side of the nose presented a curiously atrophied appearance, as also the left side of the mouth and chin; and the morbid change could be traced down the same side of the neck to about the middle. Dr. Romberg gives nine comparative measurements of the two sides: those on the left were less than the corresponding on the right, in proportions varying from a fourth to a tenth. The atrophy also implicated the left tonsil, left side of the uvula, &c.

Now it is remarkable, that there was no loss of motion or sensation on the left side, and the secretion of tears, saliva, and perspiration, was not at all affected or interrupted. The only difference was, that the pulsation of the left carotid was weaker than that of the right, and was accompanied by a dull bruit not perceptible in the latter. Dr. Romberg quotes a somewhat similar case (also in a young female) from "a collection of unpublished writings," by the late Dr. Parry: in this example, the atrophy of the left side came on with a hemiplegia of the whole left half. Dr. Romberg also relates a case communicated to him by a physician at Dantzig, resembling that observed at Berlin, in all essential particulars. Motion, sensation, and cutaneous secretion were all undisturbed.

These cases are interesting problems for the neurologist, and are not capable, we think, of a very satisfactory solution. They appear to point at a class of nerves differing from the motor, sensory, and organic, or at least that portion of the organic ministering to secretion.

Cases of *anæmia* were numerous; these were presented principally in young dressmakers, or persons engaged in sedentary employments. The anæmic condition of the blood was not, however, always indicated by pallid lips and surface: in one example, a dressmaker, aged 24, the face was so florid, that the cerebral and cardiac symptoms, observed in anæmia, were at first pronounced to be dependent on disease of the heart, and the patient was treated accordingly, without any good result. On further inquiry it was found that she had been bled seventy-five times! Steel and chalybeate mineral waters were then administered with the best success. Dr. Romberg remarks, that the characteristic bellows-sound was never absent from one or other carotid in cases of this kind: but of twelve examples, in nine it was heard in the right carotid only. A thrilling could also be felt with the finger. Gastrodynia was a usual complication, and also Pica, but the morbid appetite generally preferred sour and salt matters; in two cases only was chalk or earth selected. The treatment adopted was a course of aromatic bitters and chalybeates in different forms; in obstinate cases, chalybeate baths were prescribed; two oz. of muriated tincture of iron being added to a suitable proportion of water.

Cases of *Albuminuria* are related. The first is an example of the acute form of the disease. A girl, aged 11 years, of a scrofulous constitution, was attacked with an acute pain in the loins, particularly on the right side, œdema of the lower extremities and of the face, particularly the eyelids, slight difficulty in passing urine, and nausea. The urine was clear, frothed much when shaken, and precipitated albumen on being tested by nitric acid and heat. No exanthem preceded the attack. The patient was treated by bloodletting from the loins, mercurial inunction, calomel gr. $\frac{1}{2}$, and digitalis $\frac{1}{6}$, night and morning, together with confinement to bed, and spare diet. In eight days she was quite well.

*Iodide of iron in albuminuria.* In another example, that of a dram-drinker, aged 45, with febrile symptoms, headache, vertigo, &c. and *œdema* of the legs to the knee, and of the upper parts of the neck, the syrup of the iodide of iron was given with success, gradually from one third of a grain of the salt to a grain.

In another example in a man, aged 48, similarly and as successfully treated, the following were the results of the analysis of the blood and urine:—

The Urine in Albuminuria.

| | |
|---|---|
| Specific Gravity . . . | 1·007 |
| Water . . . . | 981·51 |
| Fixed residues . . . | 18·49 |
| Composed of | |
| Salts . . . | 3·02 |
| Uric acid . . . | 0·17 |
| Urea . . . | 1·52 |
| Albumen . . . | 4·08 |
| Extractive matters . . | 9·70 |
| | 1000· |

The Blood in Albuminuria.

| | |
|---|---|
| Water. . . . | 816·8300 |
| Fibrine . . . | 6·1529 |
| Fat from the fibrin . . | 0·0071 |
| Fat from the serum . . | 2·7800 |
| Colouring matter . . | 57·9629 |
| Albumen . . . | 62·3900 |
| Extractive . . . | 50·5700 |
| Salts . . . | 3·9800 |
| | 1000·6729 |

*Condition of the kidneys in diabetes mellitus.* Only one case of this disease came under treatment. The urine was analysed by Simon, and found to contain two ounces and three drachms of sugar to each quart. Iodide of iron, with a purely animal diet, was the treatment adopted, and at first with apparent benefit. Ultimately, however, the case terminated fatally. On an examination of the body, both the pleuræ were found to contain serum; the upper lobe of both lungs contained crude tubercles, and in that of the right, there was a circumscribed cavity of the size of a hen's egg. The liver was very voluminous and congested: the spleen wasted, flaccid, and internally of a brown colour, with soft macerated portions. Both kidneys were hypertrophied, firm, congested: the ureters normal: the bladder dilated, and its coats thickened. On more minute investigation by Dr. Remak, the cortical substance of the kidneys was found hypertrophied in consequence of thickening of the tubuli uriniferi, and especially congestion of the malpighian glands, which were thus rendered visible to the naked eye. The tubuli had an opaque appearance, dependent on a deposit in the outer coat of fat-like granules. Free fat, in the form of large globules, was also strewn over the inner surface of the tubuli, and on the mucous lining of the pelvis of the kidney. The cellular tissue connecting the tubuli and malpighian glands was indurated. No trace of sugar could be detected.

Dr. Romberg very justly argues, that the changes in the kidneys and bladder were secondary, caused by the stimulus of the saccharine urine.

*Treatment of the sequelæ of scarlatina.* In simple anasarca uncomplicated with fever, the antiphlogistic diuretics, and particularly "tartarus depuratus" (bitartrate of potass) were administered. If there were febrile symptoms, and especially those of bronchitis, a small general bleeding was ordered, with infusion of digitalis, and the bitartrate of potass. In those cases in which thoracic effusion took place, this method was actively followed, conjoined with the inunction of sublimate ointment, of the value of which Dr. Romberg speaks very favorably. A drachm of sublimate is to be mixed with an ounce of lard, and the size of a hazel-nut, or of half a teaspoonful, is to be rubbed on a circumscribed spot on the chest about the size of a crown piece, every hour until the skin becomes red. Shortly bullæ, like those of pemphigus, will appear, and when these have dried up, a new spot is to be selected, and treated in a similar manner.

*Treatment of acute rheumatism.* In recent cases, general bleeding was first practised; the painful limbs enveloped in wadding or tow, to keep them at an equable temperature, and from seven to ten minims of the tincture of colchicum seeds given every two hours, combined with two scruples of sulphate of magnesia. In mild cases, this mixture was sufficient without venesection.

The remedy found by Dr. Romberg to be most efficient in shortening the duration of an acute attack, or in very obstinate chronic cases, especially those in which one joint only was affected, was the bichloride of mercury. To adults, it was administered twice or thrice a day, in doses varying from one sixth to one third of a grain; to children, the doses varied from one twelfth to one eighth. Counter-irritants were applied in conjunction with the remedy. Rheumatic periostitis was a frequent disease, and was best treated with the iodide of potassium.

*Gangrene of the mouth* in children, occurred in the Berlin Policlinic, under the two forms of Stomacace and Noma. The former was much less malignant than the latter. It began with disease of the alveolar periosteum, then the gums were affected, and lastly the teeth dropped out. Noma commenced in the cheeks, with an ashy gray speck that gave little annoyance, and was at first overlooked. Medical aid indeed was only called in, when the disease began to appear on the cheek externally. This first began with swelling, hardness, and a peculiar fatty appearance, and then a dark gangrenous spot appeared, the preliminary to perforation of the cheek. The disease would then proceed with the most frightful ravages on the face, and not sparing even the bones. Sometimes gangrene would attack other parts, as, for example, the labia pudendi of female orphan children in the orphan houses. It was usually complicated with gastric disturbance and profuse diarrhœa, but seldom with typhus fever. It usually attacked cachectic children recovering from an infantile exanthematous fever, as measles, or scarlet fever.

In the treatment of these cases of stomacace or stomatitis, an emetic was first given if the patient was not too feeble, but generally a stimulating diet with cinchona was prescribed, and locally the application of sulphate of copper (five grains to half an ounce of honey of roses) or sulphate of zinc (ten grains to half an ounce), or the hydrochloric acid combined with honey, as an application, or diluted and used as a gargle. We have found the nitrate of silver a very trustworthy remedy in stomatitis. The child should take the eighth or sixth of a grain, with two drachms of distilled water, and two or three minims of dilute nitric acid every four hours.

*Scirrhus of the œsophagus* was observed in two cases, both men, between forty and fifty years of age. The leading symptoms were, violent pains in the throat, vomiting immediately after and even during eating, acidity, and rapid marasmus. In the one case, the scirrhus was situate just above the cardia; in the other, there had been no pain felt for some months previously to death, but a most tormenting cough supervened in its stead, accompanied by the expectoration of a dark offensive matter. On a post-mortem investigation, the upper third of the thoracic portion of the œsophagus was found affected with scirrhus. There was a fistulous opening from the diseased part to the upper lobe of the right lung, to which, and also to the vertebral column, it was strongly adherent. The fistulous opening ended in a cavity in the lung about the size of a walnut, containing a carcinomatous ichor, and the whole lung seemed infiltrated. Contrary to expectation, a second scirrhous degeneration was found in the cardia, which was united to a tumour the size of a walnut, situate on the under surface of the small lobe of the liver. There was also a small tumour of stony hardness on the pancreas.

This case is interesting, as proving, that in scirrhus of the œsophagus, other parts of the body may be implicated in the disease, contrary to the opinion of Rokitanski.

In cases in which *Gastritis*, or ulceration of the stomach, was presumed to exist, an exclusively milk diet (the milch-kur) was adopted.

*Diarrhœa of infants* was treated with the nitrate of silver, as according to the following formula:

℞. Argent. Nitr. cryst. gr. ss. Solve in Aq. destill. q. s.

Dec. Rad. Salep. ℥iij. Syr. Diacodii ℥ss. M. D. S. A dessert or teaspoonful to be taken four times daily.

We never find that the German physicians write elegant prescriptions: syr. diacodii might as well we think have been left out, and the dose of the nitrate is unnecessarily small. We have already given the formula which we prefer. We can, however, corroborate the statements of Dr. Romberg, as to the efficacy of the remedy in diarrhœa, and, we would add, in the gastric irritation that sometimes accompanies dentition.

Cases of *Chronic Peritonitis* in children, consequent on tuberculous disease of the mesentery, were common. Dr. Romberg speaks highly of the following combination (invented by Heim) in incipient tabes mesenterica:

℞. Potassæ Acetatis ʒj ad ʒij; Extr. Conii macul. gr. iv.

Aquæ communis ℥iij; Syr. Papaveris ℥j. M. A small or child's spoonful to be taken four times a day.

With this were conjoined topical bleeding, and inunction with ointment compounded of the mercurial and hydriodate of potass ointments. In atrophia lactantium, and in marasmus from chronic diarrhœa, Dr. Romberg prescribed Tokay wine with the best effects. Infants took from five to ten drops thrice daily, and children from twenty to twenty-five drops. Other dietetic means of a strengthening kind were not however omitted.

Only five cases of *Croup* were treated. In one of these a membrane was found occupying the posterior surface of the right bronchus. The inner layer of that membrane, or, in other words, that portion resting against the mucous membrane, was ascertained to be made up of amorphous cystoblast cells, with somewhat round dark granules, in which two or three nuclei might be further observed. The membrane inclosing the granule was thin and dissolved readily in acetic acid. The outer and first-formed layer of the false membrane consisted of perfectly formed fibres, in which here and there a cell-granule could still be seen. Between the fibres, there were cells prolonged into a point, and united point to point. In the whole of the exuded matter, epithelium-cells were freely scattered about. Dr. Remak examined a false membrane expectorated during a fit of coughing, and found it to consist of finely organized fibrin, soluble in acetic acid, and infiltrated with mucous corpuscles.

Amongst the *diseases of the respiratory organs*, there were observed examples of *laryngismus stridulus*, from compression of the recurrent nerves by enlarged bronchial glands. *Pleuritis* was common, and was not always readily distinguished from a rheumatic affection of the thoracic muscles. *Empyema* was treated successfully with digitalis and supertartrate of potass, in combination; externally the affected side was pencilled over with tincture of iodine, the application of it being repeated so soon as desquamation took place. In some cases, the inunction of hydriodate of potass ointment was ordered.

In simple *hypertrophy of the heart* in young subjects, a seton in the region of the heart was found to be of great service. Dr. Romberg, however, cautions his readers against expecting too much from it at first; a persistence in its use for months, or even years, is necessary, especially in cardiac disease of rheumatic origin. We have occasionally prescribed this counter-irritant, and we believe with benefit, but in the first instance it has appeared to us to aggravate all the symptoms.

In *Psoriasis inveterata,* Dr. Romberg found the aqua picea, or aqua picis liquidæ, to effect a cure when all other means failed. The aqua picea was prepared by pouring a quart of cold water over a pound of pitch, and leaving it to stand for twenty-four hours in a cool place, and a "beer-glass" (about four ounces?) of the water filtered through paper, is to be taken every morning fasting, and the parts affected to be bathed with it twice or three times a day. Its use may be continued for months, the only apparent effect resulting, being slight diuresis. Six cases are related in proof of the great efficacy of this remedy in obstinate psoriasis. It is an old remedy in England, and has been found useful in ichthyosis. Its external application in the form of ointment is also well known. The facts stated by Dr. Romberg are however useful in reminding us amidst our desire for new remedies, that there is so safe and simple a remedy as this.

A very remarkable case of *Chronic Pemphigus* was treated at the Berlin dispensary. Dr. Simon had already published an analysis of the contents of the vesicles, or bullæ, describing it as pemphigus hystericum, the patient being a highly hysterical female. The attacks recurred at intervals during three years, and were doubtless periodic: this point, however, has not received Dr. Romberg's attention. The last recorded attack occurred in January, 1840, and commenced with febrile symptoms, and a sensation as if hot water was being poured over the legs. In four and twenty hours the burning sensation extended to the surface of the body: on the next day, the epigastrium was covered with red spots, and in twelve hours afterwards, bullæ appeared on the chest, generally of an oval form, and varying in size from that of a pea to a hen's egg, and even larger. One bulla, formed by the union of several minor ones, contained at least four ounces of fluid. The bullæ were developed from day to day on the body; on the face, within the month, and even in the vagina, old bullæ drying up, and fresh ones appearing. On the seventh day from the first eruption, when it was thought the disease had ceased, suddenly a new crop broke out on the face. On the tenth day, these began to dry up, but suddenly again on the fourteenth day, another eruption took place, but was principally confined to the mouth. When desquamation took place, the hair and eyebrows fell off. The analysis of the fluid in this case is given in 'Simon's Animal Chemistry,' Sydenham Society's edition, vol. ii. Tar-water appeared to be the remedy that gave the patient the most relief.

The volume concludes with a detailed history of a case of *Elephantiasis,* or *Lepra Arabum tuberculosa,* affecting the whole surface of the body, except the hands and feet. A coloured lithograph of the face of the patient is given. The disease terminated fatally with general anasarca, and effusion into the serous cavities. A dog and a horse were inoculated with blood from the patient, and with the glairy secretion from the tubercles on the skin, but without any result.

